# An Efficient Soft Set-Based Approach for Conflict Analysis

**DOI:** 10.1371/journal.pone.0148837

**Published:** 2016-02-29

**Authors:** Edi Sutoyo, Mungad Mungad, Suraya Hamid, Tutut Herawan

**Affiliations:** Department of Information Systems, University of Malaya, Pantai Valley, Kuala Lumpur, Malaysia; Southwest University, CHINA

## Abstract

Conflict analysis has been used as an important tool in economic, business, governmental and political dispute, games, management negotiations, military operations and etc. There are many mathematical formal models have been proposed to handle conflict situations and one of the most popular is rough set theory. With the ability to handle vagueness from the conflict data set, rough set theory has been successfully used. However, computational time is still an issue when determining the certainty, coverage, and strength of conflict situations. In this paper, we present an alternative approach to handle conflict situations, based on some ideas using soft set theory. The novelty of the proposed approach is that, unlike in rough set theory that uses decision rules, it is based on the concept of co-occurrence of parameters in soft set theory. We illustrate the proposed approach by means of a tutorial example of voting analysis in conflict situations. Furthermore, we elaborate the proposed approach on real world dataset of political conflict in Indonesian Parliament. We show that, the proposed approach achieves lower computational time as compared to rough set theory of up to 3.9%.

## Introduction

Decision making is an important aspect when we consider the information about the result that we choose among two or more alternatives. There are many approaches in decision making problems which have ability to handle uncertainty, such as Fuzzy set theory [1], Rough set theory [2], Vague set theory [3], Soft set theory [4], and the recent one is Hesitant Fuzzy sets [5] as a new extension of Fuzzy sets. According to Xu [6], people are usually hesitant in making a decision and in many decision making problems they irresolute for one thing or another which makes them difficult to reach a final agreement. Decision making is one of the key components to accomplish objectives in many areas, particularly in a field which obligates analyzing the conflict. Conflict analysis is one of the fields whose importance is increasing nowadays as distributed systems of computers are starting to play a significant role in the society [7]. Conflict analysis has been used as an important role in business, economic, governmental and political dispute, games, management negotiations, military operations and etc. In conflict situation, there is uncertainty about three binary relations i.e. alliance (coalition/favorable), neutrality, and against (conflict) among agents. And the main issue is that how to find a way to model uncertainty in conflict situations [8]. In recent years, many researches have presented various mathematical approaches to handle conflict analysis, including the works of [7–11]. Firstly, Pawlak [8] introduced a mathematical of conflict situations, based on three binary relation i.e. alliance, conflict and neutrality, and given the axioms for alliance and conflict relations. In the next work, Pawlak [11] outlined a conflict graph model by representing the conflict situations with discernibility, and then proposed a new approach to conflict analysis in [10].

Regarding conflict problems using rough sets, the model introduced by Deja [12] is an enhancement of the model proposed by Pawlak in [8] by adding to the model some local aspects of conflicts. Liau [13] presented some logics with semantics based on rough set theory and related notions. An *et al*. [14, 15] proposed an integration between conflict analysis rough set-based and the idea of discernibility matrix. Furthermore, they defined different types of the coalitions according to different conflict functions and threshold values. Maeda *et al*. [16] proposed a new approach of presenting expert’s knowledge with interval importance and applied it to conflict analysis. Li *et al*. [17] presented multi-agent system (MAS) conflict analysis based on rough set theory and information granule theory, as well as introduced the notion of conflict matrix, conflict membership function and rough information granule to improve graph model in [11]. Skowron [18] proposed a rough set-based requirements determination model using a conflict relation for representing requirements agreements or disagreements. Ramanna *et al*. [19] proposed a rough set-based requirements scope determination model using generalized conflict model with approximation spaces. Inuiguchi and Miyajima [20] proposed rule induction from two decision tables based on rough sets. Yao and Zhao [21] applied discernibility and indiscernibility to conflict analysis, and introduced three types of reduction, those are discernibility, indiscernibility, and discernibility-and-indiscernibility reductions. Crossingham *et al*. [22] presented an approach to optimize rough sets partition sizes using four optimization techniques, namely, genetic algorithm, particle swarm optimization, hill climbing and simulated annealing for interstate conflict. Ma *et al*. [23] outlined the general principle of collaborative design management and resolution method, based on knowledge reasoning, granularity is used to describe design rules, and a feasible distance formula between different design rules is constructed with rough set theory. Pawlak and Skowron proposed an extension approach based on rough sets to conflict analysis in e-service intelligence [24].

With the ability to handle uncertainty from the conflict data set, rough set theory has been successfully used. Another new approach in handling uncertainty is soft set theory [4]. It is proposed by Molodtsov in 1999, which can be used as a general mathematical tool for dealing with uncertainty and imprecise data. At present, works on soft set theory are making progress rapidly both in theory and practice. For theoretical contributions, there are many extensions of classical soft set theory. Jiang *et al*. [25] proposed an extension of soft set theory by using the concepts of Description Logics (DLs) to act as the parameters of soft set. Aktaş and Çağman [26] introduced the basic properties of soft sets, compared soft sets to the related concepts of fuzzy sets and rough sets and gave a definition of soft groups. Xu *et al*. [27] introduced the notion of vague soft set. Sezgin and Atagün [28] defined the notion of restricted symmetric difference of soft sets. Babitha and Sunil [29] proposed soft set relation as a sub soft set of the Cartesian product of soft sets. Babitha and Sunil [30] defined the antisymmetric relation and transitive closure of soft set relation. Alcantud [31] investigated the formal relationships among the theories of soft sets and fuzzy sets.

There are also works on soft set theory implemented in decision making, Feng *et al*. [32] proposed an adjustable approach to fuzzy soft set based decision making, and in [33] discussed the application of interval-valued fuzzy soft sets in decision making problems. Jiang *et al*. [34] proposed an adjustable approach to intuitionistic fuzzy soft sets based on decision making. Das and Kar [35] proposed an algorithm approach based on intuitionistic fuzzy soft set (IFSS) in the group decision making (GDM) which explores a particular disease reflecting the agreement of all experts. Agarwal *et al*. [36] extended the Intuitionistic Fuzzy Soft Sets (IFSS) to generalized IFSS (GIFSS) by introducing the generalization parameter to the pool of the intuitionistic fuzzy numbers (IFNs) of IFSS, and demonstrated in decision making area. Feng and Zhou [37] introduced soft set discernibility in soft sets to solve the problems of decision making. Deli and Broumi [38] presented neutroshopic soft sets for decision making, called NSM-decision making, while Deli [39] combined an interval-valued neutrosophic sets and a soft sets, called *ivn*-soft sets and implemented it on decision making problems. Maji *et al*. [40] proposed fuzzy soft sets, and Roy and Maji [41] presented an application of fuzzy soft set theory in decision making problem. Furthermore, Alcantud [42] proposed a novel approach of fuzzy soft set in decision making in the presence of multi observer input parameter data sets.

In real life many problems are imprecise in nature, classical soft set theory is not fit of effectively dealing with such issues. Majumdar and Samanta [43] introduced the concept of generalized fuzzy soft sets. Xiao *et al*. [44] proposed the concept of D-S generalized fuzzy soft set by combining Dempster-Shafer theory of evidence and generalized fuzzy soft sets. Gong *et al*. [45] proposed bijective soft set under fuzzy environment for decision system based parameters reduction. Deng and Wang [46] proposed an object-parameter approach in incomplete fuzzy soft sets for predicting unknown data. Wang and Qu [47] introduced axiomatic definitions of entropy, similarity measure and distance measure of vague soft sets. Çağman and Deli [48] defined *t*-norm and *t*-conorm of fuzzy parameterized soft sets (FP-soft sets) and investigated their properties. Çağman and Deli [49] defined means of FP-soft sets and constructed FP-soft sets on decision making methods. Deli and Çağman [50] constructed intuitionistic fuzzy parametrized soft sets (intuitionistic FP-soft sets) for decision making. Deli and Çağman [51] proposed fuzzy soft games and applied it to financial problems. Ma *et al*. [52] proposed the idea of parameter reduction of the interval-valued fuzzy soft sets.

In this paper, we present an alternative approach to handle conflict situations, based on some ideas using soft set theory. Our motivation is to improve the computational performance by the rough sets approach in handling conflict situations when determining the support, strength, certainty, and coverage of conflict situations.

In summary, the contribution of this work is described as follows:

- We propose conflict analysis based on soft set approach.
- The novelty of the proposed approach is that, unlike rough set theory that relies on decision rules, it is based on the concept of co-occurrence of parameters in soft set theory.
- We illustrate the proposed approach by means of a tutorial example of voting analysis in conflict situations.
- Comparative analysis of the propose approach and rough set-based approach in handling conflict of Indonesian political election is presented. Furthermore, we show the efficiency of our proposed approach in term of computational time to the rough set approach.

The reminder of this paper is organized as follows. In section 2, we review the basic concepts of soft set theory. Section 3 describes the analysis of rough set theory in handling conflict situations. In section 4, we present soft set approach for conflict analysis. In section 5, we present result and discussion. Finally, the conclusion of this work is given in section 6.

## Soft Set Theory

In this section, we review some basic notion of soft set theory. To avoid difficulties, one must use an adequate parameterization. Let *U* be an initial universe set and let *E* be set of parameters in relation to object in *U*. The set *P*(*U*) denote the power set of *U*. The definition of soft set is given as follows:

### Definition 2.1

(See [7]) *A pair* (*F*,*E*) *is called a soft set over U where F is a mapping given by F*:*E* → *P*(*U*).

In other words, the soft set is parameterized family of subsets of the set *U*. Every set *F*(*e*), for *e* ∈ *E* from this family may be considered as the set of *e*-elements of the soft set (*F*,*E*), or as the set of *e*-approximate elements of the soft set.

### Example 2.1

Let be a soft set (*F*,*E*) describe the “attractiveness of cars” that Mr. X is going to buy. Suppose that *U* = {*c*_1_,*c*_2_,*c*_3_,*c*_4_,*c*_5_,*c*_6_} and *E* = {*e*_1_,*e*_2_,*e*_3_,*e*_4_,*e*_5_}, where there are six cars in the universe *U* and *E* is a set of parameters, *e*_*i*_ for *i* = 1,2,3,4,5 standing for the parameters “costly”, “safety”, “style”, “performance”, and “capacity” respectively. In this example, we consider a mapping *F*:*E* → *P*(*U*) which is given by “cars(.)”, where (.) is to be filled in by one of parameters *e* ∈ *E*. Suppose that, we have the following mapping values i.e.

$$F\left(e_{1}\right)=\left\{c_{2},c_{4}\right\},$$

$$F\left(e_{2}\right)=\left\{c_{1},c_{3}\right\},$$

$$F\left(e_{3}\right)=\left\{c_{3},c_{4},c_{5}\right\},$$

$$F\left(e_{4}\right)=\left\{c_{1},c_{3},c_{5}\right\},$$

$$F\left(e_{5}\right)=\left\{c_{1},c_{6}\right\}.$$

As we can refer to the example above, the mapping *F*(*e*_3_) means car with style characteristic, whose functional value is the set {*c*_3_,*c*_4_,*c*_5_}. Thus, we can view the soft set (*F*,*E*) as collection of approximation as follows:

$$\left(F,E\right)=\left\{e_{1}=\left\{c_{2},c_{4}\right\},\;e_{2}=\left\{c_{1},c_{3}\right\},\;e_{3}=\left\{c_{3},c_{4},c_{5}\right\},\;e_{4}=\left\{c_{1},c_{3},c_{5}\right\},\;e_{5}=\left\{c_{1},c_{6}\right\}\right\}.$$

Therefore, we can easily to understand that a soft set is not a crisp set. In previous works, it has been shown that a standard soft set (*F*,*E*) can be represented as a Boolean-valued information system (*U*,*A*,*V*_[0,1]_,*f*) (See [35, 53]). Therefore, the above soft set can be represented as a Boolean-valued information system (Table 1).

**Table 1:** Tabular Representation of Soft set (*F*,*E*).

*U* / *E*	*e*_1_	*e*_2_	*e*_3_	*e*_4_	*e*_5_
*c*_1_	0	1	0	1	1
*c*_2_	1	0	0	0	0
*c*_3_	0	1	1	1	0
*c*_4_	1	0	1	0	0
*c*_5_	0	0	1	1	0
*c*_6_	0	0	0	0	1

Soft set theory can also be used to handle multi-valued information systems and in the following section, we present the notion of multi soft sets representing multi-valued information systems.

## Multi-Soft Sets

Herawan and Deris proposed the idea of multi soft sets to for representing multi-valued information systems [54]. The idea comes from the decomposition of a multi-valued information system *S* = (*U*,*A*,*V*,*f*) to |*A*| number of Boolean-valued information systems which is based on decomposition of *A* = {*a*_1_,*a*_2_,…,*a*_|*A*|_} into a single attribute {*a*_1_},{*a*_2_},…,{*a*_|*A*|_}. In the following sub-section, we recall the construction of multi soft sets.

### Decomposition of Multi-valued Information Systems

In this section, we only consider for complete multi-valued information systems. Let *S* = (*U*,*A*,*V*,*f*) be a multi-valued information system such that for every *a* ∈ *A*, *f*(*U*,*A*) is a finite non-empty set and for every *u* ∈ *U*,|*f*(*u*,*a*)| = 1. For every *a*_*i*_ under *i*^*th*^-attribute consideration, *a*_*i*_ ∈ *A* and *v* ∈ *V*_*a*_, we define the mapping $a_{v}^{i}:U\to \left\{0,1\right\}$ such that

$$a_{v}^{i}\left(u\right)=\{\begin{array}{l} \;1,\;\text{if}\;f\left(u,a\right)=v\\ 0,\;\text{otherwise} \end{array},\;\text{for every}\;u\in U.$$

**Fig 1:**
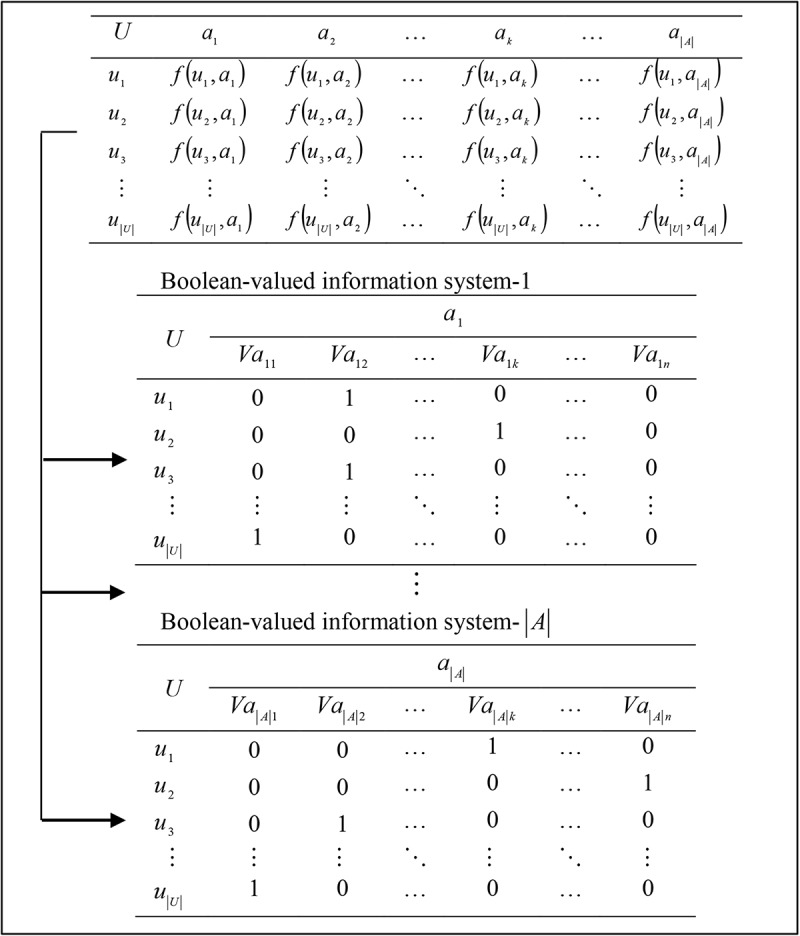
A decomposition of information systems.

The next step, we define a Boolean-valued information system as a quadruple *S*^*i*^ = (*U*,*a*_*i*_,*V*_{0,1}_,*f*). The information systems *S*^*i*^ = (*U*,*a*_*i*_,*V*_{0,1}_,*f*),1≤*i*≤|*A*| is referred to as a decomposition of a multi-valued information system *S* = (*U*,*A*,*V*,*f*) into |*A*| number of Boolean-valued information systems, as depicted in Fig 1.

Based on Fig 1, the definition of an information system, and a soft set, in this sub-section we show that a soft set is a special type of information systems, i.e., a Boolean-valued information system. The relation between a soft set and a Boolean-valued information system is given as follows:

**Proposition 3.1.** *If* (*F*,*A*) *is a soft set over the universe U*, *then* (*F*,*A*) *is a Boolean-valued information system S =* (*U*,*A*,*V*_{0,1}_,*f*).

From Proposition 3.1, each Boolean-valued information system *S*^*i*^ = (*U*,*a*_*i*_,*V*_{0,1}_,*f*), for 1≤*i*≤|*A*| in Fig 1 is a deterministic information system i.e. for every attribute *a* ∈ *A* and for every object *u* ∈ *U*, the |*f*(*u*,*a*)| is a total function. Hence, the structure of multi-valued information system and |*A*| number of Boolean-valued information systems give the same value of attribute related to objects.

### Multi-Soft Set

From sub-section 3.1, in this sub-section we present the notion of multi-soft set representing multi-valued information systems. Let *S* = (*U*,*A*,*V*,*f*) be a multi-valued information system and *S*^*i*^ = (*U*,*a*_*i*_,*V*_{0,1}_,*f*),1≤*i*≤|*A*| be a |*A*| Boolean-valued information systems. Then we have the following multi-soft sets

$$S=\left(U,A,V,f\right)=\left\{ \begin{array}{l} S^{1}=\left(U,a_{1},V_{\left\{0,1\right\}},f\right)\Leftrightarrow \left(F,a_{1}\right)\\ S^{2}=\left(U,a_{2},V_{\left\{0,1\right\}},f\right)\Leftrightarrow \left(F,a_{2}\right)\\ \;\vdots \;\vdots \;\vdots \\ S^{\left|A\right|}=\left(U,a_{\left|A\right|},V_{\left\{0,1\right\}},f\right)\Leftrightarrow \left(F,a_{\left|A\right|}\right) \end{array}\right.$$

$$=\left(\left(F,a_{1}\right),\left(F,a_{2}\right),\ldots ,\left(F,a_{\left|A\right|}\right)\right)$$

We further define the (*F*,*A*) = ((*F*,*a*_1_),(*F*,*a*_2_),…,(*F*,*a*_|*A*|_)) as a multi-soft sets over universe *U* representing a multi-valued information system *S* = (*U*,*A*,*V*,*f*).

## Proposed Soft Set Approach for Conflict Analysis

In this section firstly we recall the concept of conflict analysis from the point of view of rough set theory. Pawlak [10, 11] initiated the information system is a pair *S* = (*U*,*A*), where *U* and *A* are non-empty finite sets called *universe*, and the set of *attributes*, respectively. Pawlak models conflict analysis as follows:

1. Elements of *U* is called *objects (agents)*
2. The *A* is set of *attributes (issues)*.

Every attribute *a* ∈ *A* is a total function *a*:*U* → *V*_*a*_, where *V*_*a*_ is the set of values of *a*, called the *domain* of *a*; elements of *V*_*a*_ will be referred to as opinions, and *a*(*x*) is opinion of agent. The domain of each attribute is restricted to three values *V*_*a*_ = {1,0,−1} representing opinion of agents, where 1 means alliance, 0 means neutrality and −1 means conflict. In the following sub-section, we introduce an alternative soft set approach for conflict analysis.

### Conflict Representation in Multi-Soft Sets

Based on the multi-soft sets (*F*,*A*) as presented in sub-section 3.2, let *S* = (*U*,*A*,*V*,*f*) be a multi-valued information system and *S*^*i*^ = (*U*,*a*_*i*_,*V*_{0,1}_,*f*), for 1≤*i*≤|*A*| be a |*A*| decomposed Boolean-valued information systems. A conflict situation based on multi-soft sets is modelled as a (*F*,*A*) where quadruple *S*^*i*^ = (*U*,*a*_*i*_,*V*_{0,1}_,*f*), where *U* and *a*_*i*_ are non-empty finite sets, *U* is called *universe*, *a*_*i*_ is called *attributes*, *V* = ∪_*a*∈*A*_*V*_*a*_, *V*_*a*_ is the domain (values set) of attribute *a* and element of *V*_*a*_ is referred as opinions toward the issue, *f*:*U* × *A* → *V* is a total function such that *f*(*u*,*a*) ∈ *V*_*a*_, for every (*u*,*a*) ∈ *U* × *A*.

On a conflict situation, we can say that is special type of multi-soft sets, because *V*_*a*_ is restricted to three values *V*_*a*_ = {1,0,−1} meaning alliance (coalition), neutral, and conflict toward the issue, respectively. Therefore, here we have conflict model based on multi-soft sets as follows:

$$S^{i}=\left(U,a_{i},V_{\left\{0,1\right\}},f\right),1\leq i\leq 3,\;a_{i}\in A,V_{a}=\left\{1,0,-1\right\}$$

Let *S*^*i*^ = (*U*,*a*_*i*_,*V*_{0,1}_,*f*) be a multi-valued information system, and *S*^*i*^ = (*U*,*a*_*i*_,*V*_{0,1}_,*f*),1≤*i*≤3, *a*_*i*_ ∈ *A*, *V*_*a*_ = {1,0,−1} be a conflict model Boolean-valued information systems with *V*_*a*_ is restricted to three values *V*_*a*_ = {1,0,−1} or *V*_*a*_ = {+,0,−}. Therefore, here we have

$$S=\left(U,A,V,f\right)=\left\{ \begin{array}{l} S^{1}=\left(U,a_{i},V_{\left\{0,1\right\}},f\right)\left\{ \begin{array}{l} S^{1_{+}}=\left(U,+,V_{\left\{0,1\right\}},f\right)\Leftrightarrow \left(F,+\right)\\ S^{1_{0}}=\left(U,0,V_{\left\{0,1\right\}},f\right)\Leftrightarrow \left(F,0\right)\\ S^{1_{-}}=\left(U,-,V_{\left\{0,1\right\}},f\right)\Leftrightarrow \left(F,-\right) \end{array}\right.\\ S^{2}=\left(U,a_{j},V_{\left\{0,1\right\}},f\right)\left\{ \begin{array}{l} S^{2_{+}}=\left(U,+,V_{\left\{0,1\right\}},f\right)\Leftrightarrow \left(F,+\right)\\ S^{2_{0}}=\left(U,0,V_{\left\{0,1\right\}},f\right)\Leftrightarrow \left(F,0\right)\\ S^{2_{-}}=\left(U,-,V_{\left\{0,1\right\}},f\right)\Leftrightarrow \left(F,-\right) \end{array}\right.\\ \;\vdots \;\vdots \;\vdots \\ S^{\left|A\right|}=\left(U,a_{\left|A\right|},V_{\left\{0,1\right\}},f\right)\left\{ \begin{array}{l} S^{{{}^{\left|A\right|}}_{+}}=\left(U,+,V_{\left\{0,1\right\}},f\right)\Leftrightarrow \left(F,+\right)\\ S^{{{}^{\left|A\right|}}_{0}}=\left(U,0,V_{\left\{0,1\right\}},f\right)\Leftrightarrow \left(F,0\right)\\ S^{{{}^{\left|A\right|}}_{-}}=\left(U,-,V_{\left\{0,1\right\}},f\right)\Leftrightarrow \left(F,-\right) \end{array}\right. \end{array}\right.$$

In the following sub-section we discuss the binary relations on agents i.e. alliance (coalition), neutrality, and conflict.

### Binary relation

Let (*F*,*A*) be a soft set representing a Boolean-valued information system *S*^*i*^ = (*U*,*a*_*i*_,*V*_{0,1}_,*f*). Based on the idea from Pawlak [24], in this sub-section we present three basic binary relations on the agents, which are, alliance (coalition), neutrality, and conflict (against) as follows:

a. Alliance (coalition)

f(x,y)=1,iff(x)×f(y)=1.

That means that, if *f*(*x*,*y*) = 1, between agents *x* and *y* have the same point of view or opinion about the issue *f* (agents *x* and *y* are allied on *f*);

b. Neutrality

f(x,y)=0,iff(x)×f(y)=0.

In the case of neutrality, if *f*(*x*,*y*) = 0, at least there is one agent *x* or *y* has neutral view to conflict issue *f* (agents *x* or *y* is neutral on *f*);

c. Conflict (against)

f(x,y)=−1,iff(x)×f(y)=−1.

Finally, in the case of conflict, if *f*(*x*,*y*) = −1, both agents have different opinions about conflict issue *f* (agents *x* and *y* are conflict on *f*).

### Some Definitions

From the fact that a standard soft set (*F*,*E*) can be represented as a Boolean-valued information system (*U*,*A*,*V*_{0,1}_,*f*), in the following definition we present the notion of similarity between two parameters in (*F*,*E*). We firstly define the notion of occurrence of parameters in soft set theory.

**Definition 4.1.** *Let* (*F*,*E*) *be a soft set over the universe U representing* (*U*,*A*,*V*_[0,1]_,*f*) *and an object u* ∈ *U*. *A parameter co-occurrence set of an object u can be defined as follows*:

coo(u)={e∈E:f(u,e)=1}.

Obviously, *coo*(*u*) = {*e*∈*E*:*f*(*e*) = 1}. The following example illustrates the Definition 4.1.

**Example 4.1.** From soft set (*F*,*E*) in Table 1, parameter co-occurrence set of all objects is given as follows:

$$\text{coo}\left(c_{1}\right)=\left\{e_{2},e_{4},e_{5}\right\},$$

$$\text{coo}\left(c_{2}\right)=\left\{e_{1}\right\},$$

$$\text{coo}\left(c_{3}\right)=\left\{e_{2},e_{3},e_{4}\right\},$$

$$\text{coo}\left(c_{4}\right)=\left\{e_{1},e_{3}\right\},$$

$$\text{coo}\left(c_{5}\right)=\left\{e_{3},e_{4}\right\},$$

$$\text{coo}\left(c_{6}\right)=\left\{e_{5}\right\}.$$

From Definition 4.1, we have the following definition of agent support.

**Definition 4.2.** *Let* (*F*,*E*) *be a soft set over the universe U and an agent u* ∈ *U*. *The support of an agent u is defined by*

supp(u)=card(coo(u))=card({e∈E:f(u,e)=1}).

From Definition 4.2, we have the following definition of rules strength.

**Definition 4.3.** *Let* (*F*,*E*) *be a soft set over the universe U representing* (*U*,*A*,*V*_[0,1]_,*f*). *The strength of a rule A*_1_ ⇒ *A*_2_ *for A*_1_,*A*_2_ ⊂ *A denoted by σ*_*x*_(*A*_1_,*A*_2_) *is defined by*

$$\sigma_{x}\left(A_{1},A_{2}\right)={\text{supp}}_{x}\left(A_{1},A_{2}\right)/|U|,for\;x\in U.$$

From Definition 4.3, we have the following definition of rules certainty.

**Definition 4.4.** *Let* (*F*,*E*) *be a soft set over the universe U representing* (*U*,*A*,*V*_[0,1]_,*f*). *The certainty of a rule A*_1_ ⇒ *A*_2_ *for A*_1_,*A*_2_ ⊂ *A denoted by cer*_*x*_*(A*_1_,*A*_2_) *is defined by*

$$\begin{array}{l} {\text{cer}}_{x}\left(A_{1},A_{2}\right)={\text{supp}}_{x}\left(A_{1},A_{2}\right)/|A_{1}\left(x\right)|\\ \;=\sigma_{x}\left(A_{1},A_{2}\right)/\pi \left(A_{1}\left(x\right)\right) \end{array}$$

where *π*(*A*_1_(*x*)) = |*A*_1_(*x*)| / |*U*|.

From Definition 4.4, we have the following definition of rules coverage.

**Definition 4.5.** *Let* (*F*,*E*) *be a soft set over the universe U representing* (*U*,*A*,*V*_[0,1]_,*f*). *The coverage of a rule A*_1_ ⇒ *A*_2_ *for A*_1_,*A*_2_ ⊂ *A denoted by* cov_*x*_(*A*_1_,*A*_2_) *is defined by*

$$\begin{array}{l} {\mathrm{cov}}_{x}\left(A_{1},A_{2}\right)={\text{supp}}_{x}\left(A_{1},A_{2}\right)/|A_{2}\left(x\right)|\\ \;=\sigma_{x}\left(A_{1},A_{2}\right)/\pi \left(A_{2}\left(x\right)\right), \end{array}$$

*where π*(*A*_2_(*x*)) = |*A*_2_(*x*)| / |*U*|.

Similarly, cov_*x*_(*A*_1_,*A*_2_) = *π*_*x*_(*A*_1_|*A*_2_)

**Fig 2:**
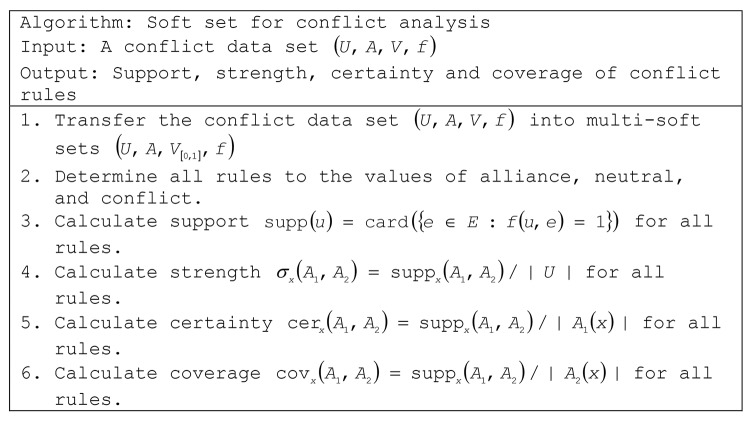
The proposed soft set-approach algorithm.

The algorithm for handling conflict data using multi-soft sets is given in Fig 2.

In the following section, we present a tutorial example of voting analysis in a conflict situation.

## Results and Discussion

In this section, we illustrate the proposed approach through an example of a conflict data set. Let a conflict situation given by a multi-valued information system (*U*,*A*,*V*,*f*) where the domain agents (universe) and the voting function *f* are respectively defined by

$$U=\left\{\begin{array}{l} \left(1,A\right),\cdots ,\left(200,A\right),\\ \left(201,B\right),\cdots ,\left(300,B\right),\\ \left(301,C\right),\cdots ,\left(500,C\right),\\ \left(500,D\right),\cdots ,\left(750,D\right)\\ \left(750,E\right),\cdots ,\left(1000,E\right) \end{array}\right\}$$

and

f(1,A)=…=f(100,A)=1,f(101,A)=…=f(130,A)=0,f(131,A)=…=f(200,A)=−1,

f(201,B)=…=f(245,B)=1,f(246,B)=…=f(280,B)=0,f(281,B)=…=f(300,B)=−1,

f(301,C)=…=f(400,C)=1,f(401,C)=…=f(500,C)=−1,

f(501,D)=…=f(650,D)=1,f(651,D)=…=f(750,D)=0,

f(751,E)=…=f(800,E)=1,f(801,E)=…=f(900,E)=0,f(901,E)=…=f(1000,E)=−1

Table 2 below is presented the conflict situation above.

**Table 2:** Conflict situation with agents (*Member*,*Party*) and the voting function Voting.

(Member, Party)	Voting	(Member, Party)	Voting
(1,*A*)	1	(451,*C*)	-1
⋯	⋯	⋯	⋯
(105,*A*)	1	(500,*C*)	-1
(106,*A*)	0	(501,*D*)	1
⋯	⋯	⋯	⋯
(135,*A*)	0	(650,*D*)	1
(136,*A*)	-1	(651,*D*)	0
⋯	⋯	⋯	⋯
(200,*A*)	-1	(750,*D*)	0
(201,*B*)	1	(751,*E*)	1
⋯	⋯	⋯	⋯
(255,*B*)	1	(800,*E*)	1
(256,*B*)	0	(801,*E*)	0
⋯	⋯	⋯	⋯
(290,*B*)	0	(870,*E*)	0
(291,*B*)	-1	(871,*E*)	-1
⋯	⋯	⋯	⋯
(300,*B*)	-1	(1000,*E*)	-1
(301,*C*)	1		
⋯	⋯		
(450,*C*)	1		

Table 2 presents a decision table in which only condition attribute is *Party*, and the decision attribute is *Voting*. The table describes voting results in a parliament containing 1000 members clustered in five political parties denoted *A*, *B*, *C*, *D* and *E*. Assume the parliament discussed particular issue and the voting result is presented in column Voting, where 1, 0 and −1 denoted alliance/coalition/favorable, neutrality and against/conflict, respectively.

From Table 2 and step 1 in Fig 2, we generate the table into multi-tables for each party based on conflict situation. For example, *A* → 1 is voted by member 1 to 105 from party *A* in which have alliance toward the issue, *A* → 0 is voted by member 106 until 135, and etc.

**Fig 3:**
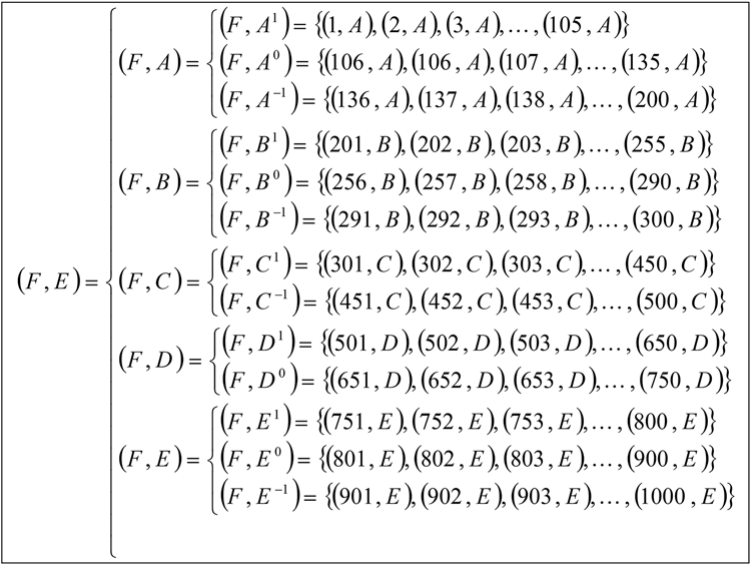
Multi-soft sets representations from Tables 3–7.

From the decomposition of a multi-valued information system (Table 2) into multi tables of Boolean-valued in Tables 3–7, we present multi-soft sets (*F*,*E*) representations as in Fig 3.

**Table 3:** A decomposition of Table 2 into multi-tables of Boolean-valued for Party A.

Member	Party A		
	1	0	−1
1	1	0	0
2	1	0	0
3	1	0	0
⋮	⋮	⋮	⋮
105	1	0	0
106	0	1	0
107	0	1	0
108	0	1	0
⋮	⋮	⋮	⋮
135	0	1	0
136	0	0	1
137	0	0	1
138	0	0	1
⋮	⋮	⋮	⋮
200	0	0	1

**Table 4:** A decomposition of Table 2 into multi-tables of Boolean-valued for Party B.

Member	Party B		
	1	0	−1
201	1	0	0
202	1	0	0
203	1	0	0
⋮	⋮	⋮	⋮
255	1	0	0
256	0	1	0
257	0	1	0
258	0	1	0
⋮	⋮	⋮	⋮
290	0	1	0
291	0	0	1
292	0	0	1
293	0	0	1
⋮	⋮	⋮	⋮
300	0	0	1

**Table 5:** A decomposition of Table 2 into multi-tables of Boolean-valued for Party C.

Member	Party C		
	1	0	−1
301	1	0	0
302	1	0	0
303	1	0	0
⋮	⋮	⋮	⋮
450	1	0	0
451	0	0	1
452	0	0	1
453	0	0	1
⋮	⋮	⋮	⋮
500	0	0	1

**Table 6:** A decomposition of Table 2 into multi-tables of Boolean-valued for Party D.

Member	Party D		
	+	0	−1
501	1	0	0
502	1	0	0
503	1	0	0
⋮	⋮	⋮	⋮
650	1	0	0
651	0	0	1
652	0	0	1
653	0	0	1
⋮	⋮	⋮	⋮
750	0	0	1

**Table 7:** A decomposition of Table 2 into multi-tables of Boolean-valued for Party E.

Member	Party E		
	1	0	−1
751	1	0	0
752	1	0	0
753	1	0	0
⋮	⋮	⋮	⋮
800	1	0	0
801	0	1	0
802	0	1	0
803	0	1	0
⋮	⋮	⋮	⋮
870	0	1	0
871	0	0	1
872	0	0	1
873	0	0	1
⋮	⋮	⋮	⋮
1000	0	0	1

From multi-soft sets (*F*,*E*) in Fig 3 and steps 2 and 3 in Fig 2, we can calculate the support of each occurrence of parameter in respected soft set by using Definition 4.3, as follows:

$$\left(F,A\right)=\left\{ \begin{array}{l} \sup\;p\left(F,A^{1}\right)=105\\ \sup\;p\left(F,A^{0}\right)=30\\ \sup\;p\left(F,A^{-1}\right)=65 \end{array}\right.$$

$$\left(F,B\right)=\left\{ \begin{array}{l} \sup\;p\left(F,B^{1}\right)=55\\ \sup\;p\left(F,B^{0}\right)=35\\ \sup\;p\left(F,B^{-1}\right)=10 \end{array}\right.$$

$$\left(F,C\right)=\left\{ \begin{array}{l} \sup\;p\left(F,C^{1}\right)=150\\ \sup\;p\left(F,C^{-}\right)=50 \end{array}\right.$$

$$\left(F,D\right)=\left\{ \begin{array}{l} \sup\;p\left(F,D^{1}\right)=150\\ \sup\;p\left(F,D^{0}\right)=100 \end{array}\right.$$

$$\left(F,E\right)=\left\{ \begin{array}{l} \sup\;p\left(F,E^{1}\right)=50\\ \sup\;p\left(F,E^{0}\right)=70\\ \sup\;p\left(F,E^{-1}\right)=130 \end{array}\right.$$

The result of all fact supports is given in Table 8.

**Table 8:** Support of all facts in Tables 3–7.

Fact	Party	Voting	Support
1	*A*	1	105
2	*A*	0	30
3	*A*	−1	65
4	*B*	1	55
5	*B*	0	35
6	*B*	−1	10
7	*C*	1	150
8	*C*	−1	50
9	*D*	1	150
10	*D*	0	100
11	*E*	1	50
12	*E*	0	70
13	*E*	−1	130

From Table 8, for each *party* we can see whether it is in alliance (coalition/favorable), neutrality, and against (conflict) among the agents. From steps 4, 5, and 6 in Fig 2, the strength, certainty and coverage for all facts in all parties are given in Table 9.

**Table 9:** The Strength, Certainty, and Coverage, Voting result.

Fact	Strength	Certainty	Coverage
1	0.105	0.525	0.206
2	0.03	0.15	0.128
3	0.065	0.325	0.255
4	0.055	0.55	0.108
5	0.035	0.35	0.149
6	0.01	0.1	0.039
7	0.15	0.75	0.294
8	0.05	0.25	0.196
9	0.15	0.6	0.294
10	0.1	0.4	0.426
11	0.05	0.2	0.098
12	0.07	0.28	0.298
13	0.13	0.52	0.510

**Fig 4:**
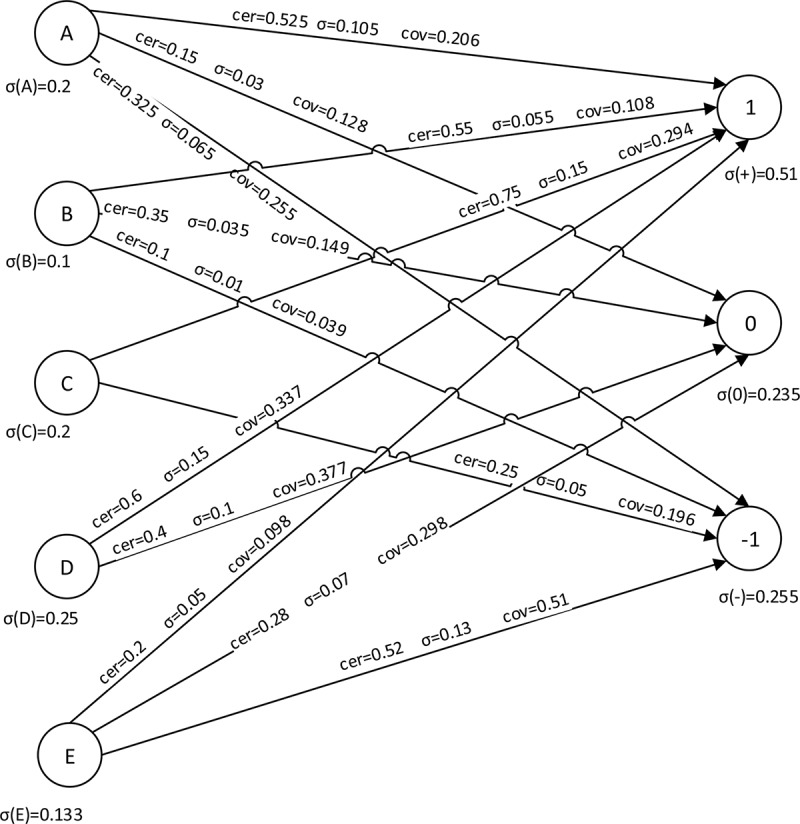
Flow graph for Table 8.

The flow graph associated with Table 9 is presented in Fig 4.

**Fig 5:**
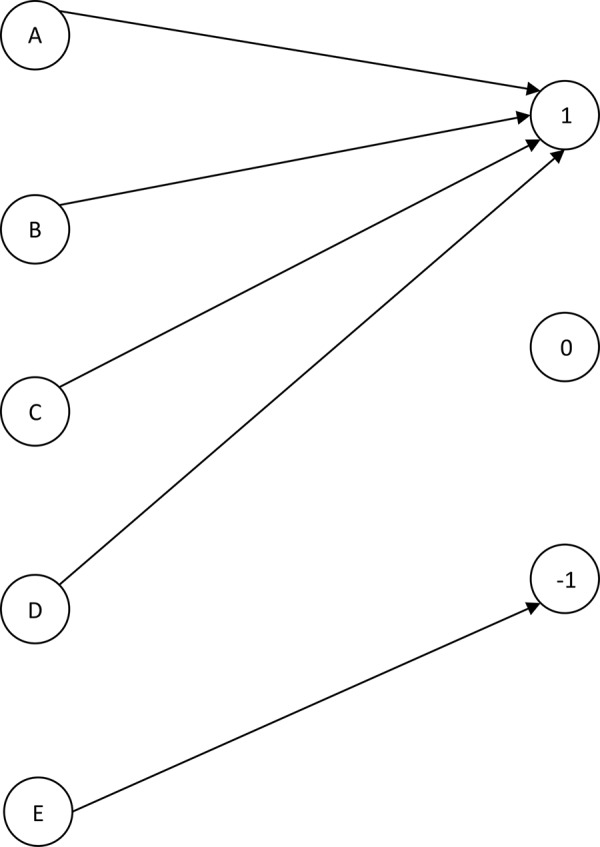
“*Approximate*” flow graph.

From Fig 4, branches of the flow graph represent point of view the agents together with their certainty and coverage factors. For instance, the *A* → 1 has the certainty factor 0.525 and coverage factor 0.206. The flow graph gives a clear insight into the voting structure of all parties. We can replace flow graph shown in Fig 4 by “approximate” flow graph shown in Fig 5.

The *“approximate”* flow graph depicted on Fig 5, we can see that parties *A*, *B*, *C*, and *D* form a coalition, which is in conflict with party *E*. This flow graph generated by using certainty factor greater than 0.5.

**Fig 6:**
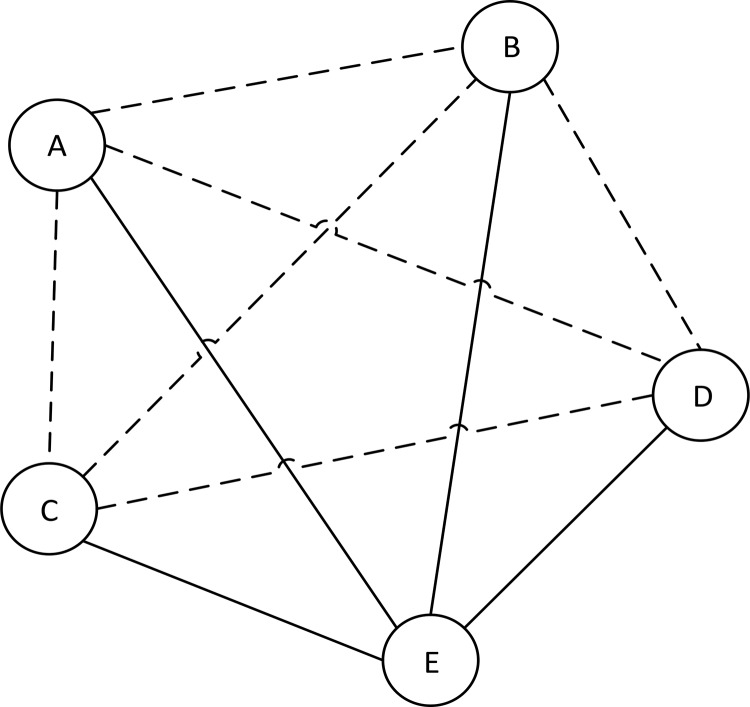
Conflict Graph.

Fig 6 shows conflict graph among all parties, solid lines are denoting conflicts and dotted lines are in alliance.

In the next section, we present an application of handling conflict in the problem of determining governor election model in Indonesia.

## Application

**Fig 7:**
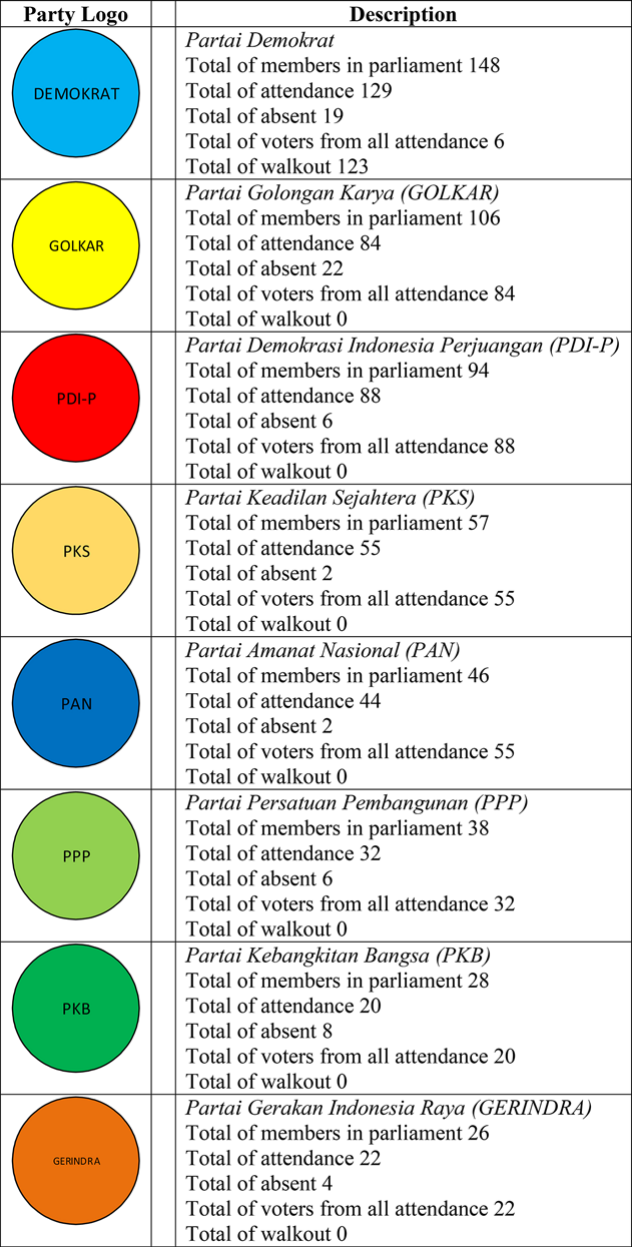
Parties Data from Parliament in Indonesia.

In this section, we present a real world application of the proposed approach to solve the problem of determining governor election model in Indonesia i.e. direct or indirect methods. The dataset is taken from liputan6 online [55] and vivanews online [56] on October 14, 2015. There are nine parties in the Indonesian parliament which is described in Fig 7.

From Fig 7, we have details information regarding the parties name and their number of members in parliament, member’s attendance, member’s absent, the total of voters from all attendance every party, and the number of member’s walkout during meeting.

Let a conflict situation given by a multi-valued information system (*U*,*A*,*V*,*f*) where the domain *ag* (universe *U*) of the voting function *f* is respectively defined by

$$U=\left\{\begin{array}{l} \left(1,\text{Demokrat}\right),\cdots ,\left(148,\text{Demokrat}\right),\\ \left(149,\text{Golkar}\right),\cdots ,\left(254,\text{Golkar}\right),\\ \left(255,\text{PDI}-\text{P}\right),\cdots ,\left(348,\text{PDI}-\text{P}\right),\\ \left(349,\text{PKS}\right),\cdots ,\left(405,\text{PKS}\right)\\ \left(406,\text{PAN}\right),\cdots ,\left(451,\text{PAN}\right)\\ \left(452,\text{PPP}\right),\cdots ,\left(489,\text{PPP}\right)\\ \left(490,\text{PKB}\right),\cdots ,\left(517,\text{PKB}\right)\\ \left(518,\text{Gerindra}\right),\cdots ,\left(543,\text{Gerindra}\right)\\ \left(544,\text{Hanura}\right),\cdots ,\left(560,\text{Hanura}\right) \end{array}\right\}$$

f(1,Demokrat)=…=f(142,Demokrat)=0,f(143,Demokrat)=…=f(148,Demokrat)=−1,

$$\begin{array}{l} f\left(149,\text{Golkar}\right)=\ldots =f\left(221,\text{Golkar}\right)=1,\;f\left(222,\text{Golkar}\right)=\ldots =f\left(243,\text{Golkar}\right)=0,\\ f\left(244,\text{Golkar}\right)=\ldots =f\left(254,\text{Golkar}\right)=-1, \end{array}$$

f(255,PDI - P)=…=f(260,PDI - P)=0,f(261,PDI - P)=…=f(348,PDI - P)=−1,

f(349,PKS)=…=f(403,PKS)=1,f(404,PKS)=…=f(405,PKS)=0,

f(406,PAN)=…=f(449,PAN)=1,f(450,PAN)=…=f(451,PAN)=0,

f(452,PPP)=…=f(483,PPP)=1,f(484,PPP)=…=f(489,PPP)=0,

f(490,PKB)=…=f(497,PKB)=0,f(498,PKB)=…=f(517,PKB)=−1,

f(518,Gerindra)=…=f(539,Gerindra)=1,f(540,Gerindra)=…=f(543,Gerindra)=0,

f(544,Hanura)=…=f(550,Hanura)=0,f(551,Hanura)=…=f(560,Hanura)=−1,

Table 10 below presents the political conflict situation above.

**Table 10:** Conflict situation with agents (*Member*,*Party*) and the voting function voting.

(Member, Party)	Voting	(Member, Party)	Voting
(1,Demokrat)	0	(449,PAN)	1
⋯	⋯	(450,PAN)	0
(142,Demokrat)	0	⋯	⋯
(146,Demokrat)	−1	(451,PAN)	0
⋯	⋯	(452,PPP)	1
(148,Demokrat)	−1	⋯	⋯
(149,Golkar)	1	(483,PPP)	1
⋯	⋯	(484,PPP)	0
(221,Golkar)	1	⋯	⋯
(222,Golkar)	0	(489,PPP)	0
⋯	⋯	(490,PKB)	0
(243,Golkar)	0	⋯	⋯
(244,Golkar)	−1	(497,PKB)	0
⋯	⋯	(498,PKB)	−1
(254,Golkar)	−1	⋯	⋯
(255,PDI - P)	0	(517,PKB)	−1
⋯	⋯	(518,Gerindra)	1
(260,PDI - P)	0	⋯	⋯
(261,PDI - P)	−1	(539,Gerindra)	1
⋯	⋯	(540,Gerindra)	0
(348,PDI - P)	-1	⋯	⋯
(349,PKS)	1	(543,Gerindra)	0
⋯	⋯	(544,Hanura)	0
(403,PKS)	1	⋯	⋯
(404,PKS)	0	(550,Hanura)	0
⋯	⋯	(551,Hanura)	-1
(405,PKS)	0	⋯	⋯
(406,PAN)	1	(560,Hanura)	-1
⋯	⋯		

Table 10 presents a decision table in which only condition attribute is *Parties with their total members in parliament*, and the decision attribute is *Voting*. The Table 10 describes voting results in Indonesian parliament containing 560 members clustered in nine political parties as described in Fig 7. Assume the parliament discussed particular issue and the voting result is presented in column *Voting*, where 1, 0 and −1 denoted *yes* (alliance/coalition/favorable), *abstention* (neutrality), and *no* (against/conflict), respectively.

From Table 10, we generate the table into multi-tables of Boolean-valued for each party based on conflict situation (See Tables 11–19). For example, Golkar → 1 is voted by member 149 to 221, and etc.

**Table 11:** A decomposition of Table 10 into multi-tables of Boolean-Valued for Demokrat.

Member	Demokrat		
	1	0	−1
1	0	1	0
2	0	1	0
3	0	1	0
⋮	⋮	⋮	⋮
142	0	1	0
143	0	0	1
144	0	0	1
145	0	0	1
⋮	⋮	⋮	⋮
148	0	0	1

**Table 12:** A decomposition of Table 10 into multi-tables of Boolean-Valued for Golkar.

Member	Golkar		
	1	0	−1
149	1	0	0
150	1	0	0
151	1	0	0
⋮	⋮	⋮	⋮
221	1	0	0
222	0	1	0
223	0	1	0
224	0	1	0
⋮	⋮	⋮	⋮
243	0	1	0
244	0	0	1
245	0	0	1
246	0	0	1
⋮	⋮	⋮	⋮
254	0	0	1

**Table 13:** A decomposition of Table 10 into multi-tables of Boolean-Valued for PDI-P.

Member	PDI-P		
	1	0	−1
255	0	1	0
256	0	1	0
257	0	1	0
⋮	⋮	⋮	⋮
260	0	1	0
261	0	0	1
262	0	0	1
263	0	0	1
⋮	⋮	⋮	⋮
348	0	0	1

**Table 14:** A decomposition of Table 10 into multi-tables of Boolean-Valued for PKS.

Member	PKS		
	1	0	−1
349	1	0	0
350	1	0	0
351	1	0	0
⋮	⋮	⋮	⋮
403	1	0	0
404	0	1	0
405	0	1	0

**Table 15:** A decomposition of Table 10 into multi-tables of Boolean-Valued for PAN.

Member	PAN		
	1	0	−1
406	1	0	0
407	1	0	0
408	1	0	0
⋮	⋮	⋮	⋮
449	1	0	0
450	0	0	1
451	0	0	1

**Table 16:** A decomposition of Table 10 into multi-tables of Boolean-Valued for PPP.

Member	PPP		
	1	0	−1
452	1	0	0
453	1	0	0
454	1	0	0
⋮	⋮	⋮	⋮
483	1	0	0
484	0	1	0
485	0	1	0
486	0	1	0
⋮	⋮	⋮	⋮
489	0	1	0

**Table 17:** A decomposition of Table 10 into multi-tables of Boolean-Valued for PKB.

Member	PKB		
	1	0	−1
490	1	0	0
491	1	0	0
492	1	0	0
⋮	⋮	⋮	⋮
497	1	0	0
498	0	1	0
499	0	1	0
500	0	1	0
⋮	⋮	⋮	⋮
517	0	1	0

**Table 18:** A decomposition of Table 10 into multi-tables of Boolean-Valued for Gerindra.

Member	Gerindra		
	1	0	−1
518	1	0	0
519	1	0	0
520	1	0	0
⋮	⋮	⋮	⋮
539	1	0	0
540	0	1	0
541	0	1	0
542	0	1	0
⋮	⋮	⋮	⋮
543	0	1	0

**Table 19:** A decomposition of Table 10 into multi-tables of Boolean-Valued for Hanura.

Member	Hanura		
	1	0	−1
544	0	1	0
545	0	1	0
546	0	1	0
⋮	⋮	⋮	⋮
550	0	1	0
551	0	0	1
552	0	0	1
553	0	0	1
⋮	⋮	⋮	⋮
560	0	0	1

**Fig 8:**
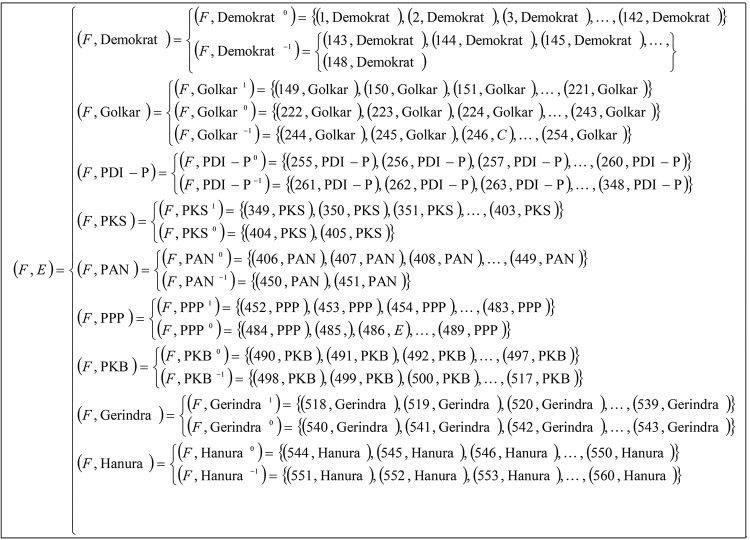
Multi-soft sets representing Indonesian parties voting (Tables 11–19).

From the decomposition of a multi-valued information system (Table 10) into multi tables of Boolean-valued in Tables 11–19, we present multi-soft sets (*F*,*E*) representations as in Fig 8.

From multi-soft sets (*F*,*E*) in Fig 8, we can calculate the support of each occurrence of parameter in the respective soft set by using Definition 4.3, as follows:

$$\left(F,\text{Demokrat}\right)=\left\{ \begin{array}{l} s\text{upp}\left(F,{\text{Demokrat}}^{0}\right)=142\\ \text{supp}\left(F,{\text{Demokrat}}^{-1}\right)=6 \end{array}\right.$$

$$\left(F,\text{Golkar}\right)=\left\{ \begin{array}{l} \text{supp}\left(F,{\text{Golkar}}^{1}\right)=73\\ \text{supp}\left(F,{\text{Golkar}}^{0}\right)=22\\ \text{supp}\left(F,{\text{Golkar}}^{-1}\right)=11 \end{array}\right.$$

$$\left(F,\text{PDI}-\text{P}\right)=\left\{ \begin{array}{l} \text{supp}\left(F,\text{PDI}-{\text{P}}^{1}\right)=6\\ \text{supp}\left(F,\text{PDI}-{\text{P}}^{-}\right)=88 \end{array}\right.$$

$$\left(F,\text{PKS}\right)=\left\{ \begin{array}{l} \text{supp}\left(F,{\text{PKS}}^{1}\right)=55\\ \text{supp}\left(F,{\text{PKS}}^{0}\right)=2 \end{array}\right.$$

$$\left(F,\text{PAN}\right)=\left\{ \begin{array}{l} \text{supp}\left(F,{\text{PAN}}^{1}\right)=44\\ \text{supp}\left(F,{\text{PAN}}^{0}\right)=2 \end{array}\right.$$

$$\left(F,\text{PPP}\right)=\left\{ \begin{array}{l} \text{supp}\left(F,{\text{PPP}}^{1}\right)=32\\ \text{supp}\left(F,{\text{PPP}}^{0}\right)=6 \end{array}\right.$$

$$\left(F,\text{PKB}\right)=\left\{ \begin{array}{l} \text{supp}\left(F,{\text{PKB}}^{0}\right)=8\\ \text{supp}\left(F,{\text{PKB}}^{-1}\right)=20 \end{array}\right.$$

$$\left(F,\text{Gerindra}\right)=\left\{ \begin{array}{l} \text{supp}\left(F,{\text{Gerindra}}^{1}\right)=22\\ \text{supp}\left(F,{\text{Gerindra}}^{0}\right)=4 \end{array}\right.$$

$$\left(F,\text{Hanura}\right)=\left\{ \begin{array}{l} \text{supp}\left(F,{\text{Hanura}}^{1}\right)=7\\ \text{supp}\left(F,{\text{Hanura}}^{0}\right)=10 \end{array}\right.$$

In the experiment, the proposed soft set-based approach is implemented in Matlab version 7.6.0.324 (R2008a). It is executed sequentially on a processor Intel Core i3 CPUs. The total main memory is 4GB and the operating system is Windows 10. The experimental results on all party supports are given in Table 20.

**Table 20:** Support of all facts of Indonesian Parties in Fig 8.

Fact	Party	Voting	Support
1	Demokrat	0	142
2	Demokrat	−1	6
3	Golkar	1	73
4	Golkar	0	22
5	Golkar	−1	11
6	PDI-P	0	6
7	PDI-P	−1	88
8	PKS	1	55
9	PKS	0	2
10	PAN	1	44
11	PAN	0	2
12	PPP	1	32
13	PPP	0	6
14	PKB	0	8
15	PKB	−1	20
16	Gerindra	1	22
17	Gerindra	0	4
18	Hanura	0	7
19	Hanura	−1	10

The strength, certainty, and coverage, of Indonesian parties voting result are given in Table 21.

**Table 21:** The Strength, Certainty, and Coverage, of Indonesian Parties Voting result.

Fact	Strength	Certainty	Coverage
1	0.254	0.959	0.414
2	0.011	0.041	0.017
3	0.130	0.689	0.160
4	0.039	0.208	0.090
5	0.020	0.104	0.044
6	0.011	0.064	0.028
7	0.157	0.936	0.393
8	0.098	0.965	0.224
9	0.004	0.035	0.015
10	0.079	0.957	0.223
11	0.004	0.043	0.019
12	0.057	0.842	0.196
13	0.011	0.158	0.068
14	0.014	0.286	0.123
15	0.036	0.714	0.300
16	0.039	0.846	0.180
17	0.007	0.154	0.066
18	0.013	0.412	0.178
19	0.018	0.588	0.247

**Fig 9:**
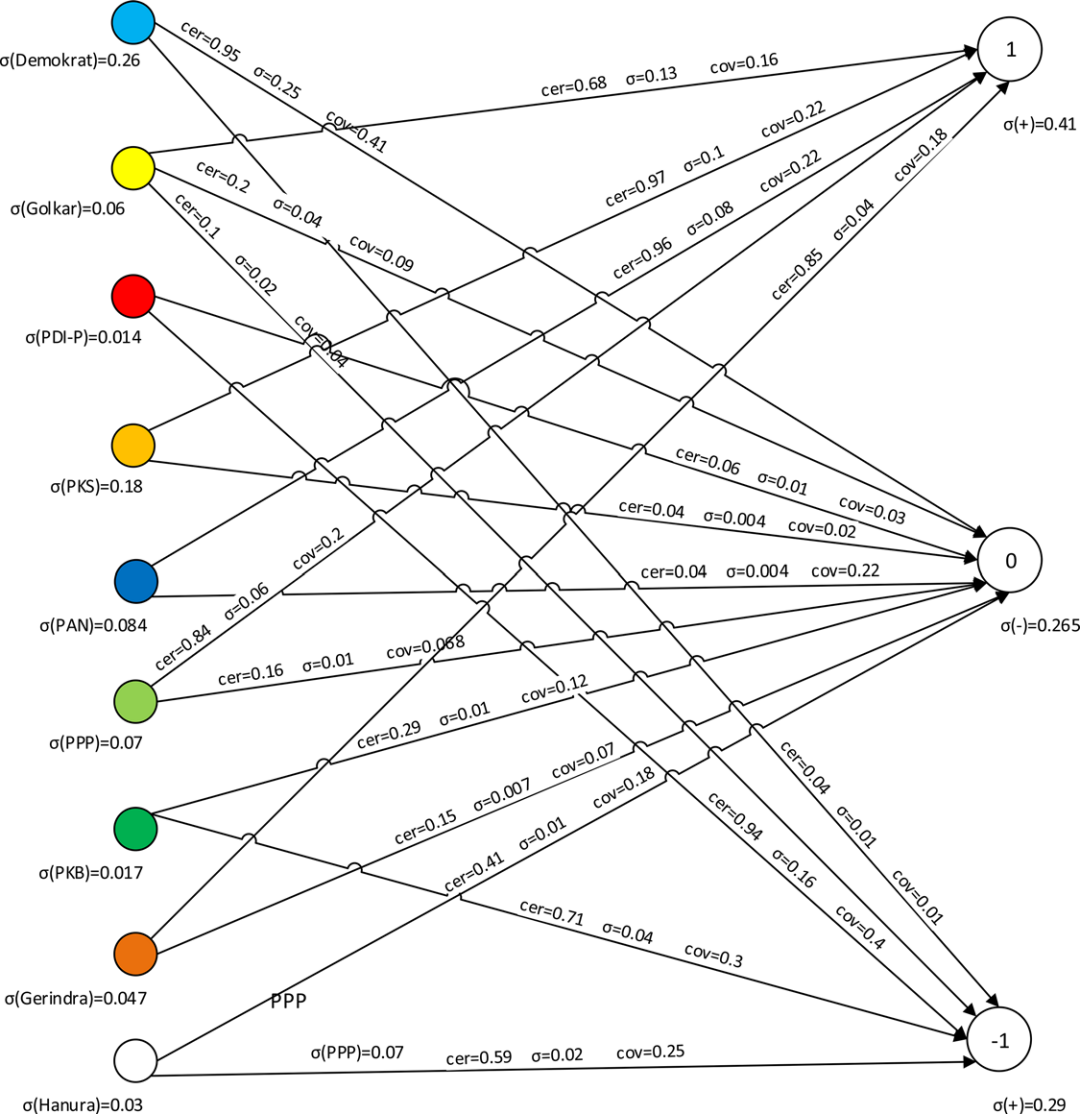
Flow graph for Table 21.

The flow graph associated with Table 21 is presented in Fig 9.

**Fig 10:**
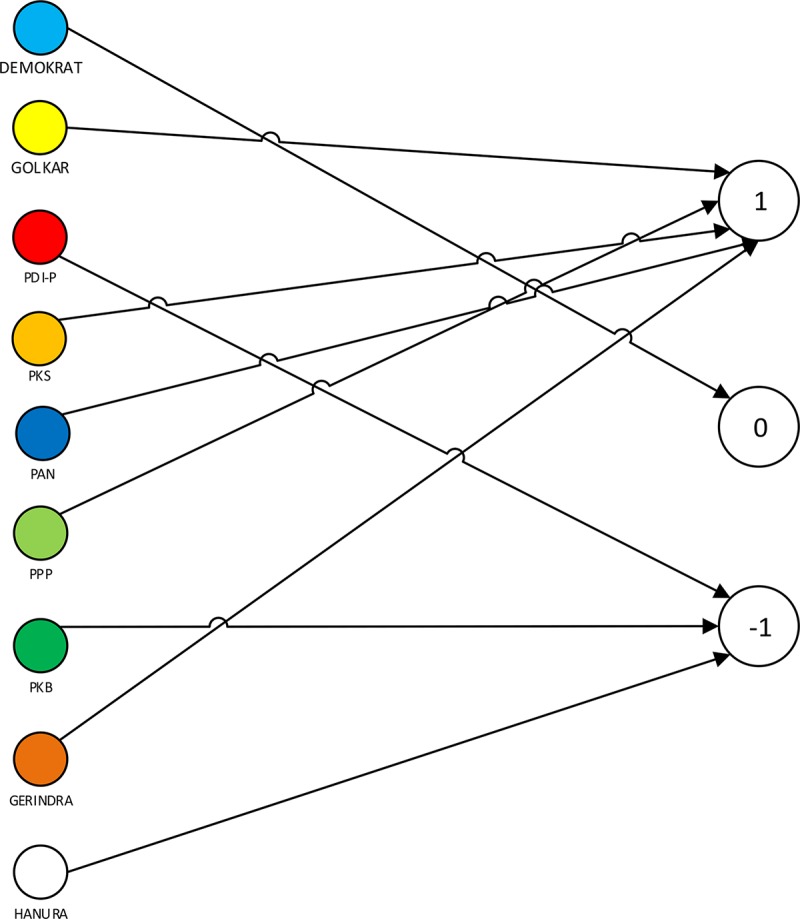
*“Approximate”* Flow Graph of Indonesian Parties.

From Fig 9, branches of the flow graph represent points of view the parties together with their strength, certainty, and coverage factors. For instance, the Golkar → 1 has the certainty factor 0.68 and coverage factor 0.16. The flow graph gives a clear insight into the voting structure of all parties. We can replace flow graph shown in Fig 9 by “approximate” flow graph shown in Fig 10.

In the “approximate” flow graph depicted on Fig 10, we can see that parties Golkar, PKS, PAN, PPP, and Gerindra form a coalition. Another group for opposition is formed by PDI-P, PKB and Hanura which is in conflict with the first coalition group. Meanwhile, Demokrat is a neutral party. This flow graph in Fig 10 is generated by using certainty factor greater than the threshold of 0.5.

**Fig 11:**
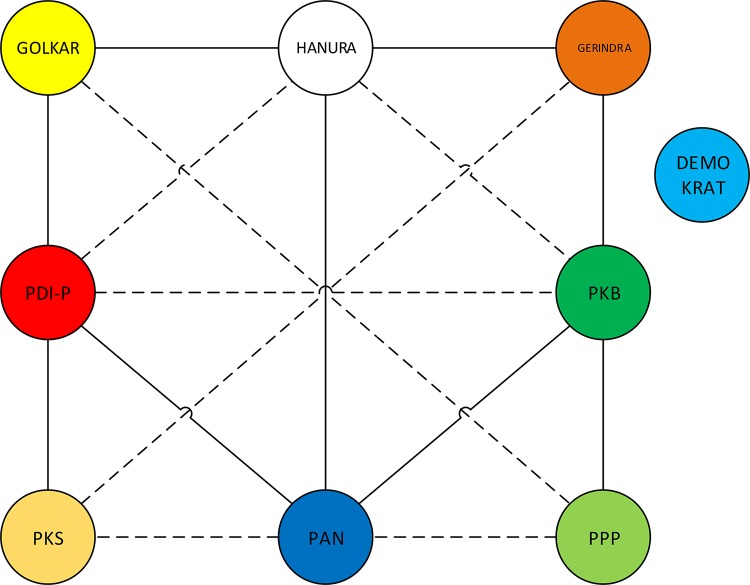
Conflict Graph.

Fig 11 above shows conflict graph among all Indonesian parties, solid lines are denoting conflicts, dotted lines are in alliance, and party which is not connected to other parties is neutral, for simplicity, in this case is Demokrat. Thus, in this case the final decision regarding governor election model in Indonesia i.e. indirect method.

The following graphs present the comparison of execution time between the proposed soft set based approach and rough set based approach on computing support, strength, certainty, and coverage.

**Fig 12:**
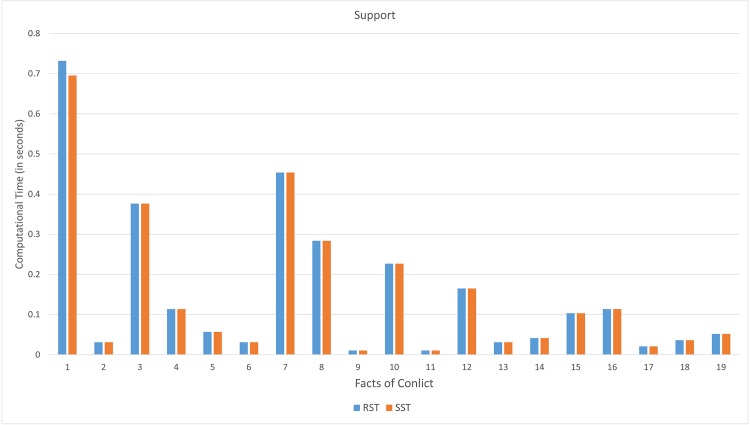
Computational time on computing supports.

From Fig 12, the computational time (in seconds) on computing supports of the proposed soft set approach tends to be lower than rough set approach. The improvement of this case is up to 1.3%.

**Fig 13:**
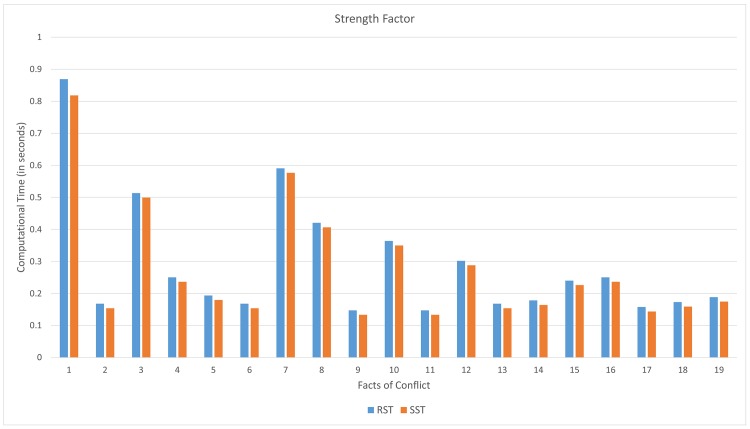
Computational time on computing strength.

From Fig 13, the computational time (in seconds) on computing supports of the proposed soft set approach tends to be lower than that rough set approach. The improvement of this case is up to 5.8%.

**Fig 14:**
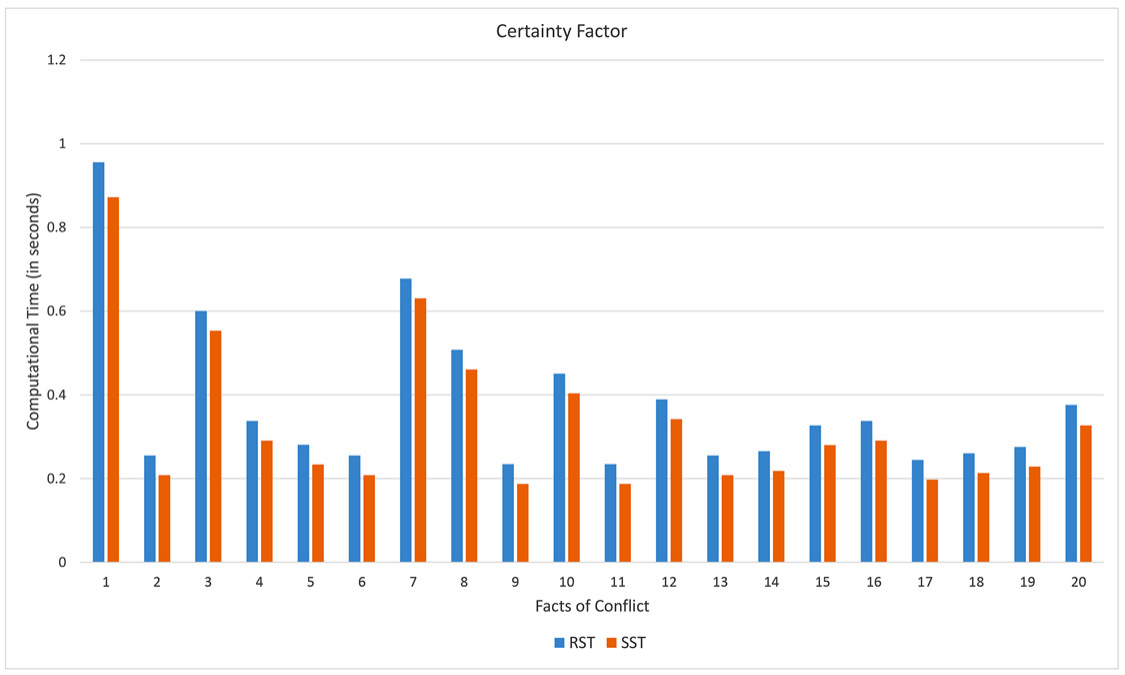
Computational time on computing certainty.

From Fig 14, the computational time (in seconds) on computing supports of the proposed soft set approach tends to be lower than that rough set approach. The improvement of this case is up to 15%.

**Fig 15:**
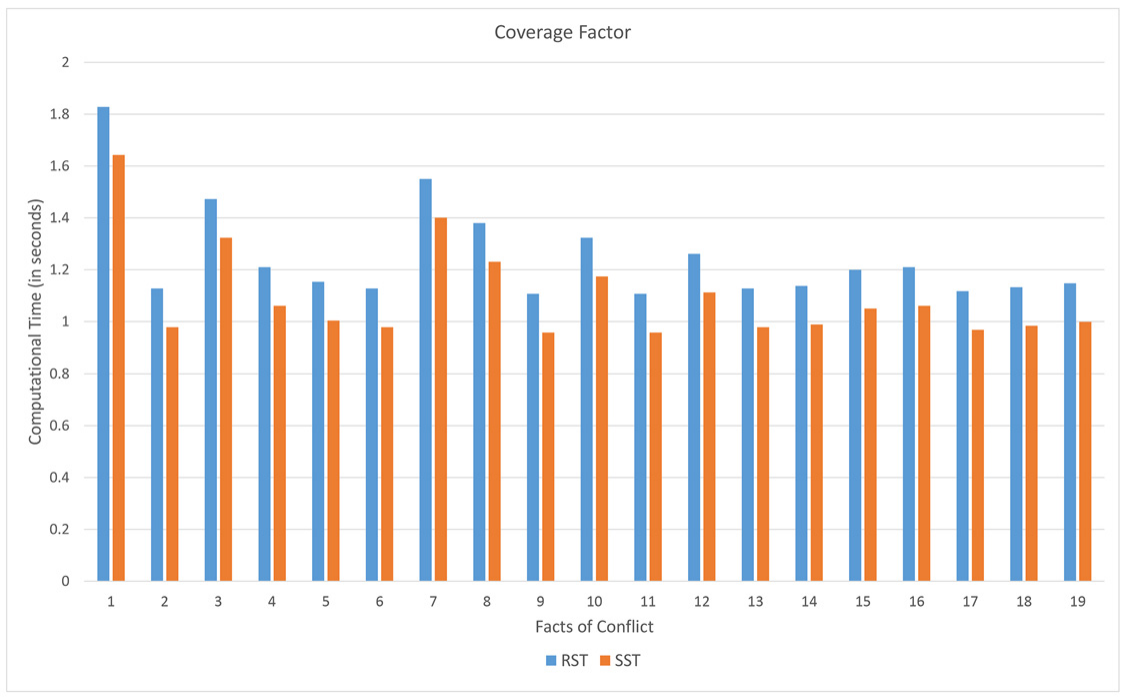
Computational time on computing coverage.

From Fig 15, the computational time (in seconds) on computing coverage of the proposed soft set approach tends lower than that rough set approach. The improvement of this case is up to 13.8%.

## Conclusion

Conflict analysis has been used as an important tool in economic, business, governmental and political dispute, games, management negotiations, military operations and etc. In this paper we have presented an alternative approach for handling conflict situation involving uncertainty. It is based on multi-soft sets taking into account of co-occurrence of parameter related to object in universe. The novelty of the proposed approach is that, unlike in rough set theory that uses decision rules, it is based on the concept of co-occurrence of parameters in soft set theory. We have presented an illustrative example on how to handle conflict using multi soft sets. Furthermore, we elaborate the proposed approach of real world dataset of voting from political election data set from Indonesian parliament. However, we achieve lower computational time as compared to rough set approaches. In the future work, we will extend this proposed soft set-based method by refinement of the neutrosophic set [57, 58] and its application to other area of conflict, such as urban planning and business.
